# Impact of premature rupture of membranes on clinical outcomes of extremely premature infants: A propensity score matching study

**DOI:** 10.3389/fped.2023.1144373

**Published:** 2023-03-30

**Authors:** Jing-Ke Cao, Chang-Geng Liu, Dan Wang, Qiu-Ping Li

**Affiliations:** ^1^ The Second School of Clinical Medicine, Southern Medical University, Guangzhou, China; ^2^ Department of Neonatology, Senior Department of Pediatrics, The Seventh Medical Center of PLA General Hospital, Beijing, China

**Keywords:** extremely premature infants, premature rupture of membranes (PROM), propensity score matching (PSM), disease prognosis, complications

## Abstract

**Background:**

Premature rupture of membranes (PROM) is a common cause of extremely premature infants (EPIs) and also leads to adverse preterm complications. However, the effect of PROM on EPIs remains contradictory. This study used propensity score matching (PSM) to adjust the baseline characteristics to explore the impact of PROM on clinical outcomes of extremely premature infants (EPIs).

**Methods:**

Medical data of 470 EPIs at gestational age < 28weeks who received prenatal examination in our hospital between January 1, 2015 and December 31, 2020 were analyzed retrospectively. According to the presence or absence of PROM, they were divided into a PROM group and a non-PROM group. Ten covariates including birth weight, male sex, artificial conception, cesarean delivery, 5-min Apgar score ≤ 7, oligohydramnios, gestational hypertension, preeclampsia, antenatal steroid use, and complete steroid treatment were matched 1:1 by PSM. The major complication occurrence and mortality during hospitalization were compared between the two groups by *t-test*, nonparametric test or $x^{2}$ test.

**Results:**

Among the 470 infants enrolled, 157 (33.4%) were in the PROM group and 313 in the no-PROM group. After matching the ten confounding factors,276 cases were successfully enrolled. The incidence of early pulmonary hypertension (EPH) and severe retinopathy of prematurity (ROP) in the PROM group were higher than those in the no-PROM group [44.2% (61/138) vs. 29.0% (40/138); 34.8% (48/138) vs. 21.7% (30/138), *x*^2^ = 6.886 and 5.790, both *P* < 0.05]. However, there was no significant difference in the in-hospital mortality and the incidence of other major complications between the two groups (all *P* > 0.05).

**Conclusions:**

PROM increased the incidence of EPH and severe ROP in EPI, but had no significant impact on in-hospital mortality, length of hospital stay, and the incidence of other complications.

## Introduction

Premature rupture of membranes (PROM) is a common complication during pregnancy, and its incidence is about 5%–10% (1). According to the time of onset, PROM can be classified as full-term PROM and preterm premature rupture of membranes (PPROM). The earlier the time of occurrence, the greater the potential harm. About one-third of premature births are related to PROM (1, 2). In particular, PPROM, which usually occurs before the gestational age of 28 weeks, increases the incidence of extremely premature infants (EPIs) (3). Due to the immaturity of various organs, the prognosis of these infants is relatively poor, and the incidence of complications such as severe bronchopulmonary dysplasia (BPD), necrotizing enterocolitis (NEC), and intraventricular haemorrhage (IVH) is high. However, the extent of adverse impact of PROM in EPIs remains uncertain. Some studies reported that the occurrence of PPROM increased the incidence and even mortality of diseases such as respiratory distress syndrome (RDS), sepsis, BPD, severe retinopathy of prematurity (ROP), IVH and NEC, among others (4, 5), while others held different views, believing that survival rates and serious complications in these infants were similar to those of preterm infants without PROM (4, 6, 7). These different results may be due to differences in study participants and baseline characteristics. In addition, there are few research data on EPIs. In this retrospective study, we analyzed the clinical data of EPIs who were admitted to the Department of Pediatrics of the 7th Medical Center of the PLA General Hospital (Beijing, China), compared the baseline characteristics of the patients in the PROM group and the non-PROM group by the propensity score matching (PSM) method after eliminating the influence of individualized factors, and explored the relationship of PROM with complications and mortality in EPIs during hospitalization for the sake of providing clinical basis for better prenatal consultation about neonatal care and management of EPIs born from mothers with PROM.

## Material and methods

### Study design

In this retrospective study, we selected EPIs at a gestational age < 28 weeks who were admitted to the Department of Pediatrics of the 7th Medical Center of the PLA General Hospital between January 1, 2015 and December 31, 2020. Exclusion Criteria (1) postnatal time >72 h; (2) incomplete birth or hospitalization information; (3) serious genetic or inherited metabolic diseases; and (4) abandonment of treatment due to social factors. According to the presence or absence of PROM before birth, they were divided into a PROM group and a non-PROM group.

### Methods

Data collection: Perinatal and neonatal clinical data of the newborns were collected by reviewing the inpatient medical records. Perinatal data included the amniotic fluid contamination, oligohydramnios, fetal distress, gestational hypertension, gestational diabetes, preeclampsia, placental factors, prenatal infection, antenatal steroid use, and complete steroid use. Clinical data of the neonates included gestational age, birth weight, male sex, multiple births, artificial conception, cesarean delivery, 1 min Apgar score ≤ 7, 5 min Apgar score ≤ 7, surfactant therapy, death during hospital stay, neonatal hospital stay, RDS, BPD, moderate and severe BPD, early pulmonary hypertension (EPH), bronchopulmonary dysplasia associated with pulmonary hypertension (BPD-PH), duration of invasive mechanical ventilation, days of oxygen use, pneumonia, early-onset sepsis (EOS), late-onset sepsis (LOS), patent ductus arteriosus (PDA), PDA ligation, IVH, severe IVH, periventricular leukomalacia (PVL), NEC, ROP, and severe ROP.

### Definitions

Placental factors included placental abruption, placenta previa, and placenta accrete. Death during the hospital stay refers to the failure of rescue efforts during hospitalization, or the abandonment of treatment due to the terminal stage of the disease. The diagnosis and classification criteria of BPD are as follows: (1) oxygen inhalation for at least 28 days; and (2) infants who did not need oxygen and were evaluated at 36 weeks of postmenstrual age (PMA) or at discharge. Those who needed oxygen but with FiO_2 _< 0.3 were considered moderate cases, and who needed oxygen but with FiO_2_ ≥ 0.3 or needed positive pressure ventilation were considered severe cases (8). EPH and BPD-PH: According to echocardiography, the specific diagnostic criteria refer to the diagnosis of pulmonary hypertension in premature infants (9), and the PH diagnosed within 3–14 days after delivery of EPH. The presence of PH in children with BPD after 36 PMA was referred to as BPD-PH. EOS was defined as septicemia when the clinical manifestations were observed within 72 h after birth, while LOS was defined as sepsis when the clinical manifestations were observed 72 h after birth (10). IVH was graded according to Papile's criteria (11). Grade III IVH referred to bilateral ventricular enlargement with ventricular hemorrhage, and grade IV referred to intraventricular hemorrhage with periventricular hemorrhagic infarction, both of which were considered severe forms of IVH. NEC referred to Bell stage ≥ II (12). ROP was diagnosed and staged according to the international diagnostic and staging standards of ROP (13). Severe ROP referred to stage ≥ III and required laser or *Ranibizumab* treatment.

### Statistical analysis

All statistical analyses were performed using SPSS 26.0 statistical software. Measurement data of normal distribution are represented as $\bar{X}$ ±s, and two-sample t-test was used for the between-group comparison. Measurement data of abnormal distribution are represented by M (P25, P75), and the *Mann-Whitney U* test was used for nonparametric test for intergroup comparison. The number of use cases and percentage (%) of the counting data indicate that the $x^{2}$ test was used for the between-group comparison. The PSM method was used to match 1:1, and the caliper value was 0.01. Variable selection for the PSM model: baseline parameters with statistically significant differences between groups were selected as covariates for score matching, knowing that these covariates may affect the clinical outcomes of EPIs. *P* < 0.05 was considered statistically significant.

## Results

### Comparison of the two sets of basic conditions before and after matching

A total of 470 patients were enrolled, including 157 in the PROM group and 313 in the non-PROM group. The time of premature rupture of membranes was 48(18.5,96.0) hours before propensity matching. After matching, it was 48(18.3,96.0) hours. The basic situation before and after matching of the two groups is shown in Tables 1, 2. Before matching, there were 10 covariate differences between the two groups (all *P* < 0.05), including birth weight, male sex, artificial conception, cesarean delivery, 5 min Apgar score ≤ 7, oligohydramnios, gestational hypertension, preeclampsia, antenatal steroid use, and complete steroid treatment. After matching, 276 EPIs were successfully matched, and all covariates matched well, and the differences were not statistically significant (all *P* > 0.05).

**Table 1:** Characteristics of the extremely premature infants.

Characteristics	Before matching				After matching			
	PROM (*n* = 157)	non-PROM (*n* = 313)	$x^{2}$/Z/*t* value	*P* value	PROM (*n* = 138)	non-PROM (*n* = 138)	$x^{2}$/Z/*t* value	*P* value
GA^a^, wk	27 (27.3,26.2)	27 (27.3,26)	−0.194	0.846	26.8 (26,27.2)	27 (26.2,27.4)	−1.529	0.126
Birth weight^b^,g	996.6 ± 155.9	951.8 ± 204.7	−2.636	0.009	989.6 ± 160.8	1013.7 ± 194.1	1.121	0.263
Male sex	79 (50.3%)	125 (39.9%)	4.588	0.032	66 (47.8%)	60 (43.5%)	0.526	0.468
Multiple birth	59 (37.6%)	98 (31.3%)	1.848	0.174	50 (36.2%)	44 (31.9%)	0.581	0.446
Artificial conception	99 (63.1%)	226 (72.2%)	4.101	0.043	47 (34.1%)	44 (31.9%)	0.148	0.701
Cesarean delivery	32 (20.4%)	101 (32.3)	7.280	0.007	27 (19.6%)	25 (18.1%)	0.095	0.758
1 min Apgar ≤7	71 (45.2%)	155 (49.5%)	0.774	0.379	65 (47.1%)	55 (39.9%)	1.474	0.225
5 min Apgar ≤ 7	30 (19.1%)	86 (27.5%)	3.938	0.047	28 (20.3%)	22 (15.9%)	0.879	0.248
Surfactant therapy	146 (93.0%)	296 (94.6%)	0.463	0.496	127 (92.0%)	129(93.5%)	0.216	0.642

^a^ *Z* value.

^b^ *t* value; PROM, premature rupture of membranes.

**Table 2:** The characteristics of the pregnant mothers.

Characteristics	Before matching				After matching			
	PROM (*n* = 157)	non-PROM (*n* = 313)	$x^{2}\;$value	*P* value	PROM (*n* = 138)	non-PROM (*n* = 138)	$x^{2}$ value	*P* value
Amnio fluid contamination	13 (8.3%)	29 (9.3%)	0.125	0.724	12 (8.7%)	9 (6.5%)	0.464	0.496
Oligohydramnios	15 (9.6%)	14 (4.5%)	4.663	0.031	5 (3.6%)	9 (6.5%)	1.204	0.273
Fetal distress	18 (11.5%)	32 (10.2%)	0.169	0.681	15 (10.9%)	10 (7.2%)	1.100	0.294
Gestational diabetes	34 (21.7%)	68 (21.7%)	<0.001	0.986	30 (21.7%)	27 (19.6%)	0.199	0.656
Gestational hypertension	5 (3.2%)	63 (20.1%)	24.254	<0.001	5 (3.6%)	6 (4.3%)	0.095	0.758
Preeclampsia	7 (4.5%)	31 (9.3%)	4.172	0.041	6 (4.3%)	3 (2.2%)	1.034	0.309
Placental factors	28 (17.8%)	77 (24.6%)	2.759	0.097	24 (17.4%)	32 (23.2%)	1.434	0.231
Prenatal infection	40 (25.5%)	62 (19.8%)	1.978	0.160	38 (27.5%)	37 (26.8%)	0.018	0.892
Antenatal steroids	116 (73.9%)	191 (61.0%)	7.637	0.006	93 (67.4%)	90 (65.2%)	0.146	0.702
Complete steroids treatment	77 (49.0%)	109 (34.8%)	8.842	0.003	67(48.6%)	56(40.6%)	1.775	0.183

### Comparison of clinical outcomes between the two groups

Before matching: The incidence of BPD, EPH, BPD-PH and early-onset sepsis during hospitalization in the PROM group was higher than that in the non-PROM group (*P* < 0.05), and there was no significant difference in the incidence of mortality, neonatal hospital stay, RDS, BPD, duration of Invasive mechanical ventilation, days of oxygen use, pneumonia, LOS, PDA, IVH, severe IVH, PVL, NEC, ROP and severe ROP between the two groups (*P* > 0.05) (Table 3).

**Table 3:** Clinical outcomes of the extremely premature infants.

Clinical outcomes	Before matching				After matching			
	PROM (*n* = 157)	non-PROM (*n* = 313)	$x^{2}/Z$ value	*P* value	PROM (*n* = 138)	non-PROM (*n* = 138)	$x^{2}/Z$ value	*P* value
Death during hospital stay	17 (10.8%)	54 (17.3%)	3.365	0.067	17 (12.3%)	14 (10.1%)	0.327	0.567
Neonatal hospital stay^a^, day	81 (64.5,98.5)	78 (20.3,99.8)	−0.939	0.348	81 (57.5,98)	73 (32.8,94)	−1.442	0.149
RDS	154 (98.1%)	303 (96.8%)	0.641	0.423	136 (98.6%)	133 (96.4%)	1.319	0.251
BPD	129 (82.2%)	230 (73.5%)	4.370	0.037	110 (79.7%)	105 (76.1%)	0.526	0.468
Middle-severe BPD	95 (60.5%)	164 (52.4%)	2.782	0.095	80 (58.0%)	75 (54.3%)	0.368	0.544
EPH	67 (42.7%)	103 (32.9%)	4.321	0.038	61 (44.2%)	40 (29.0%)	6.886	0.009
BPD-PH	16 (10.2%)	15 (4.8%)	4.974	0.026	10 (7.2%)	4 (2.9%)	2.709	0.100
Duration of Invasive mechanical ventilation, d	10 (3,27)	10 (3,23)	−0.359	0.719	10 (3.8,27.0)	9 (2,20)	−1.268	0.205
Days of oxygen use	10.0 (0,21.0)	12 (4.0,21.5)	−1.491	0.136	10 (0,20.3)	12 (4.0,21.5)	−1.428	0.153
Pneumonia	73 (46.5%)	139 (44.4%)	0.184	0.668	66 (47.8%)	77 (55.8%)	1.756	0.185
EOS	14 (8.9%)	12 (3.8%)	5.170	0.023	12 (8.7%)	7 (5.1%)	1.413	0.235
LOS	25 (15.9%)	31 (9.9%)	3.610	0.057	21 (15.2%)	12 (8.7%)	2.788	0.095
PDA	130 (82.8%)	251 (80.2%)	0.464	0.496	113 (81.9%)	111 (80.4%)	0.095	0.758
PDA ligation	8 (5.1%)	24 (7.7%)	1.090	0.296	8 (5.8%)	9 (6.5%)	0.063	0.802
IVH	87 (55.4%)	177 (56.5%)	0.055	0.815	79 (57.2%)	79 (57.2%)	<0.001	>0.999
Severe IVH	29 (18.5%)	74 (23.6%)	1.634	0.201	27 (19.6%)	26 (18.8%)	0.023	0.879
PVL	18 (11.5%)	26 (8.3%)	1.229	0.268	17 (12.3%)	18 (13.0%)	0.033	0.856
NEC	29 (18.5%)	53 (16.9%)	0.172	0.679	24 (17.4%)	27 (19.6%)	0.216	0.642
NEC operation	11 (7.0%)	23 (7.3%)	0.018	0.893	9 (6.5%)	12 (8.7%)	0.464	0.496
ROP	75 (47.8%)	132 (42.2%)	1.330	0.249	66 (47.8%)	59 (42.8%)	0.717	0.397
Severe ROP	54(34.4%)	88(28.1%)	1.956	0.162	48(34.8%)	30(21.7%)	5.790	0.016

^a^ *Z* value; RDS, respiratory distress syndrome; BPD, bronchopulmonary dysplasia; PH, pulmonary hypertension; BPD-PH, bronchopulmonary dysplasia associated with pulmonary hypertension; EOS, early-onset sepsis; LOS, lately-onset sepsis; PDA, patent ductus arteriosus; IVH intraventricular haemorrhage; PVL, periventricular leukomalacia; NEC, necrotizing enterocolitis; ROP, retinopathy of prematurity.

After matching: The incidence of EPH and severe ROP during hospitalization in the PROM group was higher than that in the non-PROM group (*P* < 0.05), and there was no significant difference in the incidence of other complications between the two groups (*P* > 0.05), and there was no significant difference in mortality and length of hospital stay between the two groups (*P* > 0.05) (Table 3).

## Discussion

In this review article, we explored the impact of PROM on clinical outcomes of EPIs by PSM analysis, and found that the incidence of EPH and severe ROP increased in the PROM group as compared with that in the non-PROM group, while there was no clear difference in mortality and length of hospital stay between the two groups.

Current findings on PROM for EPI survival and length of hospital stay reported in the literature are inconsistent (14, 15). A previous study (14) reported that PROM increased the mortality in preterm infants compared with other settings. Gezer et al. (16) believed that the mortality rate of children with PROM was mainly related to preterm birth, and the younger the gestational age at birth, the higher the mortality rate. Newman et al. (17) reported that PROM increased the infant mortality at 23–24 weeks, but they did not find a significant relationship between the infant mortality and PROM after adjusting for gestational age and sex. Two other studies (18, 19) suggested that birth weight was the strongest risk factor for survival and length of hospital stay. In our study, we did not find significant differences in in-hospital mortality and length of hospital stay between the two groups before and after matching, which is consistent with the finding of Yair et al. (15) and Hanke et al. (7), who reported that PROM did not increase the EPI in-hospital mortality in mothers of the same gestational age and birth weight.

Some studies (5, 20, 21) suggested that PROM increased the occurrence of EOS and NEC, but the baseline data on oligohydramnios and antenatal steroid treatment in these studies were inconsistent. In addition, cesarean section and 5 min Apgar scores have also been shown to be associated with the occurrence of NEC and EOS (22–24). We did not find significant differences in the incidence of EOS and NEC between the two groups after matching these factors, but we found that the incidence of EPH in the PROM group was higher than that in the non-PROM group both before and after matching, which is similar to previous studies (6). PH was found to be closely related to the mortality of preterm infants, BPD and long-term cardiopulmonary diseases, and a recent study (6) reported that EPH after long-term PROM increased the mortality and IVH in preterms. Data concerning EPH in preterm infants are limited, and it is usually associated with oligohydramnios secondary to PROM because the swelling pressure formed by the lung fluid in the airways is the main physical force that stimulates normal lung development, and oligohydramnios can reduce the size of the thoracic cage and interfere with the normal growth of the fetal lung (25). This may explain our finding why the incidence of EPH in the PROM group was higher than that in non-PROM group before matching, but the incidence of EPH in the PROM group was still higher than that in non-PROM group after adjusting for factors such as oligohydramnios, indicating that PROM increased the incidence of EPH, which we believe may be related to pulmonary dysplasia complicated by PROM. At present, it is known that PROM can participate in the pathogenesis of pulmonary dysplasia *via* various mechanisms such as inflammatory factors, abnormal expression of pulmonary surfactant protein, vascular endothelial growth factor (VEGF) gene polymorphisms and oxidative stress (26). Early injury to the developing lung impairs angiogenesis and alveolar formation, leading to simplified distal lung spaces and resulting in PH (27). In addition, PROM has also been shown to accelerate fetal lung maturation, increase the degree of pulmonary artery musculosis, and increase incidence of EPH in preterm neonates (28).

ROP is a common complication of EPIs and a leading cause of blindness in children (29). Preterm birth and postpartum oxygen therapy have been reported to be closely related to the occurrence of ROP, but many studies have proposed that the pathogenesis of ROP may exist *in utero*, and the prenatal intrauterine environment may be related to the occurrence of severe ROP (30, 31), including maternal preeclampsia (32), chorioamnionitis (33), and smoking (34). In addition, gender has also been shown to be associated with the occurrence of severe ROP (35). At present, there are conflicting reports about the relationship between PROM and ROP. Lynch et al. (36) and Badeeb et al. (37) showed that PROM was a protective factor for severe ROP, possibly because pregnant mothers who develop PROM are more likely to receive glucocorticoids, which plays an important role in promoting the maturation of the cerebrovascular system by downregulating VEGF to improve angiogenesis and systemic circulation (38). But when we matched these factors, we found that the incidence of severe ROP was higher in the PROM group than that in the non-PROM group. Another study (39) also concluded that newborns with PROM longer than 18 h had an increased risk of severe ROP. We think that this may be because the inflammatory-oxidative stress axis involved in PROM is also involved in ROP formation (40).

To clarify the impact of PROM on mortality and complications during EPI hospitalization, we used the PSM method to match the baseline data of EPIs between the two groups and found that PROM increased the incidence of EPH and severe ROP during EPI hospitalization. Nevertheless, the study also has some limitations. First, this is a non-randomized retrospective study, which may miss some unobserved differences such as some treatment measures and data on chorioamnionitis, antenatal magnesium use and delayed cord clamping due to imperfect obstetric pathological data. That could have an impact on the results. Second, this study only discussed the short-term prognosis, and did not assess the long-term prognosis, especially the occurrence of BPD-PH after discharge. Finally, this study is a single-center study with a relatively small sample size. More data from multiple centers are required to further validate the conclusions of this study.

In summary, antenatal PROM increased the incidence of EPH and severe ROP during EPI hospitalization, but had no significant impact on mortality, length of hospital stay, and other morbidities during hospitalization. For EPIs appearing in the second trimester of pregnancy, more and special attention should be paid to the screening of postnatal PH and ROP, and timely prevention and treatment measures should be given to help improve the prognosis of EPIs. Our data may provide some information about the quality and outcomes of EPI treatment in mothers with PROM, and provide clinical guidance to help improve the quality of treatment for EPIs and future research.

## Data Availability

The original contributions presented in the study are included in the article/supplementary material, further inquiries can be directed to the corresponding author/s.
